# How Canada Changed from Exporting Asbestos to Banning Asbestos: The Challenges That Had to Be Overcome

**DOI:** 10.3390/ijerph14101135

**Published:** 2017-09-27

**Authors:** Kathleen Ruff

**Affiliations:** Senior Human Rights Adviser, Rideau Institute, Ottawa, ON K1P 5A6, Canada; kruff@bulkley.net

**Keywords:** asbestos trade, asbestos lobby, distortion of science, public health advocacy, international solidarity campaign

## Abstract

Less than ten years ago, the asbestos industry enjoyed the support of every Quebec and Canadian political party. The Chrysotile Institute and the International Chrysotile Association, both located in Quebec, aggressively marketed asbestos around the world, claiming scientific evidence showed that chrysotile asbestos could be safely used. The industry created a climate of intimidation. Consequently, no groups advocating for victims of asbestos or campaigning for its outright ban existed in Quebec to challenge the industry. A campaign was launched to mobilize the scientific community to speak out. Working with scientists, activists, and asbestos victims around the world, a small group of Quebec scientists exposed the false arguments of the asbestos industry. They publicly and repeatedly challenged the unscientific and unethical asbestos policy of the government. By appealing to Quebec values and holding those in power accountable, the campaign won public support and succeeded against all odds in defeating the asbestos industry.

## 1. Introduction

Until recently, Canada was a major producer, exporter and propagandist for the asbestos industry. Other Western countries banned asbestos many years ago, but it was only in December 2016 that the government of Canada finally announced that it would ban asbestos by 2018 [1].

In order to achieve an asbestos ban in Canada, it was critical to understand the political, economic, social, cultural, and historical factors that underlay Canada’s policy of supporting the mining, use, and export of asbestos. A key factor was that Quebec had for more than a century been the heart of the asbestos industry’s power. While asbestos mining had shut down in all other parts of Canada, asbestos mining continued in Quebec and the last two mines operating there—the Jeffery mine in the town of Asbestos and the LAB Chrysotile mine at the town of Thetford Mines—were moving forward in 2012 with plans to expand the mining and export of asbestos for the next twenty years. They had the full support of the Quebec and Canadian governments.

In September 2012, a new minority Parti Québécois government was elected in Quebec. It cancelled a $58 million loan that the previous Jean Charest government had given in June 2012 to finance the expansion of the Jeffery mine. Thus, the political and financial support that, for decades, Quebec governments had given to the asbestos industry ended. Both asbestos mines closed down. All mining and export of asbestos ceased.

The Quebec asbestos industry had historically enjoyed the support of every political party at the provincial and national level. By the 1980s, in the face of indisputable scientific evidence of asbestos harm and epidemics of asbestos-related deaths in industrialized countries, the asbestos industry faced a crisis. It foresaw that its traditional customers in the West would soon ban or stop buying asbestos.

In order to save the asbestos industry, in 1984 the Canadian and Quebec governments, together with the owners of the Quebec asbestos mines and Quebec trade unions, established and financed the Asbestos Institute in Montreal. Its mission was to create new markets for asbestos in developing countries. In 1997, the Asbestos International Association, whose Directors represented asbestos mines in Quebec, Russia, Kazakhstan, Brazil, China, and Zimbabwe, as well as asbestos industrialists in India, Indonesia, Vietnam, Thailand, Sri Lanka, and Mexico, moved from France to Montreal to work hand in hand with the Asbestos Institute. In 2005, in a public relations move to avoid using the word “asbestos”, they changed their names to the Chrysotile Institute and the International Chrysotile Association.

Because Canada has a good international reputation and is seen as a scientifically and democratically advanced country, Canada’s image was of enormous value to the global asbestos trade.

## 2. Status Analysis

### 2.1. Key Stakeholders

As stated in the title of a renowned Quebec novel [2], Canada consists of two solitudes—Quebec and the rest of Canada—each with its own language, history, culture and legal system. The battle against the asbestos industry had to be won in Quebec. Under Canada’s constitutional arrangements, trade issues fall under federal jurisdiction, but mining is governed by provincial jurisdiction. Beyond this jurisdictional division, however, was the profound cultural reality that defeating the Quebec asbestos industry could only be achieved through a campaign that was respectful of Quebec values and sensitivities, that understood Quebec’s political and social realities, that was conducted in French, and that mobilized leadership and public support in Quebec itself.

Until the asbestos industry was defeated in Quebec, there was no possibility of the Canadian government banning asbestos.

Key stakeholders who would normally play a leading role in initiating a campaign to ban asbestos are asbestos victims organizations and trade unions. This was not a possibility in Quebec. No asbestos victims organization existed in Quebec nor was there a Canadian asbestos victims organization.

The trade unions had joined the asbestos lobby. The most powerful trade union leader in Quebec, Clément Godbout, the former President of the Quebec Federation of Labour, became the President of the Chrysotile Institute and the International Chrysotile Association.

The asbestos lobby was aggressive, had financial resources, and had political and social power. A climate of intimidation existed such that no one in Quebec would launch a campaign to ban asbestos. When efforts were made to create an asbestos victims organization and hold a meeting in Thetford Mines in 2008, they were met with ugly insults and threats. Federal, provincial, and local political leaders, together with trade union and business leaders and the asbestos lobby, held a press conference denouncing the “anti-chrysotile militants” and warning them to stay away or be met by a “special reception committee” [3].

The group cancelled its meeting and ended its efforts to create a victims organization.

The situation was thus dire. It was urgent to launch a campaign to defeat the asbestos industry. If Jeffery Mine and LAB Chrysotile succeeded with their plans to vastly expand their mining activities and make Quebec the second biggest exporter of asbestos in the world, it would be much more difficult to shut down these mines once workers had been hired and once production and export increased.

To succeed, a campaign had to be launched and won in Quebec.

### 2.2. Obstacles that Had to Be Overcome

Among the difficult challenges to be addressed were the following:

- A climate of silence and intimidation existed in Quebec regarding asbestos.
- The two asbestos mines were no longer owned by powerful, distant U.S. and English Canadian corporations, but by local businessmen who lived and worked in the two communities. The workers themselves were co-owners of Jeffrey Mine Inc. through a co-operative which bought 35% of the company shares to rescue the mine from bankruptcy in 2002.
- Quebec asbestos miners were historically and culturally held in high esteem because of the heroic strike by the miners at the town of Asbestos in 1949 against the U.S. Johns Manville Company that owned the mine. Although the striking miners failed to achieve their demands, the courage displayed by the miners, their wives, and families against appalling exploitation was a turning point in sparking a movement for self-determination, justice, and pride in Quebec [4].
- The town of Asbestos was a single industry town that grew up around the asbestos mine. Thetford Mines had succeeded in diversifying its economic base but was still vulnerable. The workers and local political, union, and business leaders were, as they saw it, fighting for their jobs and the survival of their community. It was critical to not disregard the plight of the workers and instead demand that the Canadian and Quebec governments provide funding to allow workers to retire with dignity or transition to other jobs and to launch initiatives for sustainable, economic diversity for a post-asbestos era.
- The political, economic and social power of the asbestos industry in Quebec was enormous. The asbestos lobby aggressively attacked anyone who opposed asbestos as being a crazy fanatic and being paid by hidden interests. The campaign against the asbestos industry had no financial resources and virtually no support in Quebec when it began.
- The Canadian and the international asbestos lobby organizations were based in Montreal. They had financial resources, employees, consultants, lawyers, and public relations companies to promote the asbestos trade. They paid scientists large amounts of money to publish distorted research claiming that amphibole asbestos is hazardous but that chrysotile asbestos can be safely used. Chrysotile asbestos is the only kind of asbestos traded in the world today. It represents 95% of all the asbestos sold over the past century. This false “safe use of chrysotile argument”, created in Quebec, is still today the major weapon used by the asbestos industry to market asbestos and to maintain its profits at the expense of human life.

### 2.3. Positive Factors That Could Be Harnessed

Because of Quebec’s historical experience of political, linguistic, cultural, and religious oppression and discrimination, the issue of Quebec identity and national pride and the enjoyment of international respect have a particular resonance.

- In order for French language and culture to survive while surrounded by an ocean of English power, a particular sense of solidarity and social justice has taken root in Quebec.
- Scientists and academics in Quebec take pride in believing that their work and their institutions meet first class standards and deserve international respect.
- Some political parties, some individual politicians, some trade union organizations, and some individual trade union leaders were publicly committed to progressive ideals of international solidarity, human rights, scientific integrity, and worker health. If publicly challenged, it would be difficult for them to justify why they were supporting the export of asbestos overseas, why they were being complicit with deadly, corrupt scientific arguments, why they were rejecting the appeals of the world trade union movement, and why they were betraying their commitment to social justice.
- Many journalists and media outlets in Quebec take pride in doing quality journalism. If clear evidence was put before them, showing that the information the government and the asbestos industry were putting forward was false, there was the possibility of getting media coverage to expose the wrongdoing and winning public support. Of particular interest to the media and to the Quebec public would be if Quebec was receiving international attention and its conduct internationally condemned.

### 2.4. The Battle against the Quebec Asbestos Industry Begins

The key power of the Quebec asbestos industry lay in the fact that it had taken control over the scientific message. It claimed to be playing a positive role in helping developing countries by promoting “the safe, controlled use of chrysotile” and supplying a valuable product. The vast majority of the Quebec population had no understanding of the scientific evidence on chrysotile asbestos. It was therefore easy to create confusion and doubt. The asbestos lobby published glossy propaganda, bearing the official emblems of the governments of Canada and Quebec, stating that “recent scientific studies” demonstrated that chrysotile was not hazardous [5]. They did not disclose that these “scientific studies” were financed by the asbestos lobby.

It was essential to challenge the misinformation disseminated by the asbestos industry, which was the foundation of its success. It is almost always more effective to challenge an issue when there is a concrete, real life event occurring, rather than putting forward a general, abstract challenge. Such a real life event was about to occur.

In 2006, the Canadian government played the leading role in opposing the listing of chrysotile asbestos as a hazardous substance under the United Nations (UN) Rotterdam Convention. The Convention does not impose a ban; it simply requires exporting countries to obtain Prior Informed Consent from any country to which they wish to export the hazardous substance.

The next meeting of the Rotterdam Convention was to take place in September 2008. This presented an opportunity to put the spotlight on the scientifically and morally indefensible role Canada was playing in sabotaging the recommendation of the Convention’s expert scientific body to put chrysotile asbestos on the Convention’s list of hazardous chemicals.

In the lead-up to the meeting, an internet advocacy organization based in British Columbia, RightOnCanada.ca, drafted in French and English a *World Call of Conscience to Canada’s Prime Minister Stephen Harper to Stop Obstructing the Rotterdam Convention* [6]. The *Call of Conscience* stated that Canada’s sabotage of a basic human right provided by the Convention was endangering the Convention, was contrary to Canadian values, and constituted an ugly smear on Canada’s international reputation.

Efforts had been made to reach out to scientists in Quebec. Consequently, among the well over one hundred scientists from around the world who signed the *Call of Conscience* were twenty-six health experts from Quebec universities, the city of Montreal’s health authority, and Quebec government health institutions. The President of Quebec’s leading human rights organization, La Ligue des droits et libertés, was also a signer.

Thus, the climate of silence and intimidation in Quebec was cracked open. Quebec scientists, as well as a Quebec human rights leader, had publicly challenged the Canadian government’s asbestos policy at the UN. The scientific expertise and status of the signers of the letter made it impossible for the asbestos lobby to attack the signers as crazy, ignorant fanatics.

The *World Call of Conscience* received much media attention. The Chrysotile Institute lobbied hard to persuade the Canadian government to once again play the lead role in opposing the listing of chrysotile asbestos at the 2008 Rotterdam Convention Conference of the Parties. Instead, the Canadian government was silent and refused to answer any media questions, thus losing credibility.

The letter was a first, brave step by Quebec health scientists to publicly challenge the government’s asbestos policy. The Statement appealed to scientific integrity, human solidarity, and international reputation—values that had a particular resonance in Quebec.

The next action was again to challenge a concrete, current event—the $750,000 funding the Canadian government had announced it would give to the Chrysotile Institute. The Conservative government of Prime Minister Harper was particularly vulnerable on this issue as it promoted an ideological belief in free enterprise capitalism, which opposed government subsidies to industry lobby organizations.

In January 2009, two Quebec health experts, together with five English Canada signers, sent a letter to Prime Minister Harper, calling on him to cancel the $750,000 funding for the Chrysotile Institute [7]. The letter challenged the “misleading and untruthful information” disseminated by the Chrysotile Institute and called for an end to “the wasteful use of public funds which is harming Canada’s scientific and moral reputation around the world and exposes innocent people to harm from asbestos”. It asked for the funds to be used to assist the asbestos workers and provide sustainable economic development in the asbestos communities.

The letter received wide media coverage. It was a shock to the Chrysotile Institute, the government, and the Quebec public that Quebec health experts were leading an attack on the Chrysotile Institute. Neither Prime Minister Harper nor the Chrysotile Institute were able to defend the scientific and moral wrongdoing the letter documented. Both suffered a serious blow in the court of public opinion.

The momentum grew in September 2009, when fifteen prominent scientists from Quebec universities and health institutions published an Open Statement in La Presse, Quebec’s biggest circulation newspaper, challenging the Quebec government to respect scientific evidence, respect the lives of workers overseas, and stop mining and exporting asbestos [8]. The Statement underlined the overwhelming scientific consensus that chrysotile asbestos causes deadly diseases and the harm that Quebec’s asbestos was causing overseas. The call for action appealed to both the minds and the hearts of the public.

This marked a pivotal turning point. The asbestos industry’s claim that scientific evidence supported the “safe use” of chrysotile asbestos had been exposed as false in the most public way possible by Quebec’s own health experts. The scientists’ arguments focused on scientific integrity and human solidarity. “This infamy is no longer defensible”, said the scientists. “It is time to align ourselves with the truth”.

For the first time, the people of Quebec were challenged by respected Quebec figures to act truthfully and honorably and stop Quebec’s mining and export of asbestos.

The asbestos lobby had money, lawyers, public relations consultants, and political influence. The health experts who spoke up had none of these. Instead, they demonstrated integrity and courage in speaking truth to power. Their appeal resonated with Quebec values and culture. In the arena of public opinion, they brought a credibility that no money can buy.

Over and over again, the campaign mobilized scientists to speak up to and call for public policy to be based on independent, reputable scientific evidence and to protect human life. This was not done in an abstract way but by engaging in real issues, naming names and holding people accountable.

When the government of Quebec held parliamentary hearings on Quebec’s mining legislation, Quebec health experts gave a powerful presentation, stating that Quebec’s export of asbestos brought dishonour to Quebec and must stop [9].

A complaint was submitted to the Quebec College of Physicians that Quebec’s Minister of Health, Yv Bolduc, was violating his Code of Ethics as a medical doctor in supporting the use of chrysotile asbestos [10].

When Radio-Canada, Quebec’s public broadcasting commission, provided biased, inaccurate coverage of the asbestos issue, complaints were submitted to Radio-Canada’s Ombudsman. The complaints were upheld. Journalists who put forward asbestos industry misinformation learned they would be challenged and publicly discredited.

The small group of scientists who first broke the silence and challenged the asbestos industry gradually won the support of other professional bodies. The Quebec Medical Association, the Quebec Association for Public Health, the Quebec Cancer Society, the Quebec Association of Physicians specializing in Community Health, the Lung Association of Quebec, the Quebec Association for Occupational Hygiene, Health & Safety, and the Quebec College of Family Physicians all now called publicly for an asbestos ban. Not a single health organization or reputable scientist supported the asbestos industry.

In an extraordinary act of leadership, all sixteen of the Quebec government’s Directors of Public Health sent a letter stating that, if the government gave the $58 million loan to the Jeffery mine, the subsequent expansion of mining and use of chrysotile asbestos in Quebec would result in an increase in asbestos-related diseases among workers and the general population, creating social and financial costs. The Directors issued a press release and posted their letter on the Quebec government website [11,12].

The challenge of the asbestos industry was done in a public and transparent manner. Intense, prolonged media coverage put a spotlight on the asbestos industry. Sunlight is a powerful disinfectant. It became clear that the arguments of the asbestos industry lacked credibility and were rejected as nonsense by the scientific community. The industry’s use of ugly insults, calling the government health scientists “a little band of Taliban”, and calling those who opposed the asbestos industry corrupt liars no longer succeeded in intimidating anyone and was recognized as dirty tactics of a desperate, discredited industry.

Editorial after editorial was published in Quebec condemning the asbestos industry and calling on the government to stop supporting it.

### 2.5. Canada’s Minister of Health Challenged

In December 2009, Quebec and Canadian health experts, together with leading environmental and health organizations, including the Canadian Cancer Society, wrote a letter challenging Canada’s Minister of Health, Leona Aglukkaq, to respect scientific evidence, fulfill her duty to protect the health of Canadians and support a ban on asbestos [13].

Criticism of asbestos has “been a taboo for quite a while in Quebec. We’re trying to break that”, stated Pierre Gosselin, professor in the faculty of medicine at Laval University and a researcher at the National Public Health Institute of Quebec, who signed the letter. This was the first time a federal health minister had been asked to take action on asbestos, and her reaction will be seen as a litmus test for her support of public health measures, the health experts told the media.

The Minister refused to meet the health experts and could not answer their damning evidence. She was publicly exposed as failing to fulfill her duty to protect health.

### 2.6. Quebec Challenged Internationally

In January 2010, scientists around the world wrote to Quebec Premier Charest challenging Quebec to stop mining and exporting asbestos [14]. The letter documented how Quebec’s asbestos policy was scientifically and ethically indefensible and brought international shame on Quebec.

Charest was at the time embarking on a trade mission to India, accompanied by Quebec asbestos exporter, Baljit Singh Chadha, and by Normand Paulin, Quebec’s Director of Occupational Safety.

In India, asbestos victims, trade unionists, and health activists asked Charest to meet with them and learn directly about how Quebec asbestos was harming workers, their families, and communities in India. Charest refused a meeting, but the Quebec journalists accompanying Charest met with the asbestos victims. Trade unionists and activists met with Paulin, informed him how “safe use” of chrysotile asbestos was impossible to practice in India and called on him to act ethically and stop supporting the export of asbestos to India.

The appeals of the Indian asbestos victims, trade unionists, and activists for Quebec to stop exporting asbestos and harming the lives of populations overseas received major coverage on Quebec television, radio, and print media, as did the challenge of Premier Charest by the international scientific community.

The Quebec population thus received a powerful message that the international scientific community condemned Quebec’s export of asbestos as bringing dishonour on Quebec worldwide. The coverage of the protests by asbestos victims and activists in India brought home the reality of the human suffering being caused by asbestos. This coverage had a major impact on public opinion.

Challenges were not only made to the Quebec and Canadian governments but were repeatedly made to every Quebec and federal political party. Parties that expressed commitment to social justice and international solidarity were challenged to demonstrate these values by ending their support of the asbestos industry. As a result, in April 2009, Canada’s left-wing political party, the New Democratic Party, called for an end to asbestos mining and export, the first political party in the House of Commons to do so. In February 2010, Amir Khadir, the leader of Quebec Solidaire, called for the mining and export of asbestos to stop, the first political party in the Quebec National Assembly to do so.

Little by little, as a result of continuous, very public advocacy actions and growing public opposition to asbestos export, other political parties at first split on the issue and then eventually came on side to support banning asbestos. Finally, by 2012, the Conservative party in Ottawa and the Liberal party in Quebec were isolated as the only political parties supporting the asbestos industry. Each had a vested interest in supporting the asbestos industry. Charest represented the asbestos mining region. Harper was ideologically dedicated to mining interests. Unfortunately, these two political parties were the governments in power in Quebec and Canada.

### 2.7. Asia-Quebec Solidarity Delegation Comes to Quebec

In December 2010 a delegation from Asia of asbestos victims, health activists and a trade unionist came to Quebec to make a direct appeal to Quebec to reject the proposal to give the asbestos industry a $58 million government loan to allow it to vastly expand its mining and export of asbestos for the next two decades.

Supported by the leader of Québec Solidaire and a New Democratic Party Member of Parliament, as well as Quebec health experts, the delegation held press conferences in the Quebec National Assembly and the House of Commons. They met with political and union leaders, did media interviews, and held public events. Together with Quebec human rights and health activists, they held a demonstration outside the office of Premier Jean Charest.

The Solidarity delegation brought a powerful human message to Quebec. The fact that the delegation was working together with Quebec political, health, and human rights leaders was an inspiring example of international solidarity.

On the scientific and human level, the asbestos industry was increasingly discredited and losing the battle for public support.

A public opinion poll, commissioned in January 2011 by the Canadian Association of Physicians for the Environment, showed that 76 percent of Quebecers opposed government financing of the Jeffrey mine.

In March 2011, for the first time, a Quebec trade union confederation (CNTU) stopped supporting the asbestos industry. The monopoly of power that the asbestos industry had held for so long over political parties, unions, and the media was finally being broken.

By early 2012, in the face of public pressure, the Canadian government ended its 24 years of financing the Chrysotile Institute. Without government support, the Institute closed down.

On Friday, 29 June 2012, just before a special long holiday weekend and just two months before a provincial election, the Charest government gave a $58 million loan to the Jeffrey mine. While the government tried to bury the news, it was met with a storm of protest [15]. The asbestos industry had by now overwhelmingly lost public support in Quebec itself.

Just days before the election, the Parti Québécois announced that, if it was elected, it would cancel the loan [16]. The party was then elected on 4 September 2012 and proceeded to cancel the loan, and asbestos mining ceased in Quebec.

In October 2015, after almost ten years in power, the Conservative government of Stephen Harper was defeated and the Liberal Party of Justin Trudeau took power. The asbestos industry was now closed down in Quebec, and political and public attitudes in Quebec had completely turned around on the asbestos issue. In June 2016, all four political parties in the Quebec National Assembly supported a motion awarding the medal of the Quebec National Assembly to Kathleen Ruff to honour the campaign that had been carried out to end asbestos mining in Quebec, ban asbestos, and protect the health of workers and Quebec citizens [17].

In December 2016, the Trudeau federal government announced that it will pass legislation to ban asbestos by 2018 and will hold consultations to develop effective regulations and implementation.

## 3. Lessons Learned

It was important to understand the political and social foundation of the asbestos industry’s power in order to challenge it effectively. This required winning the battle of public opinion in Quebec. The campaign was fought in as open and public a way as possible. Every opportunity was seized to get media coverage and reach the minds and the hearts of the public.

The campaign was based on sound scientific evidence, but putting forward evidence in a general way is not sufficient. It was critical to carry out repeated advocacy actions that put a spotlight on concrete, current events that drew public attention, that resonated with Quebec values, and that held those who possessed political and social power publicly accountable.

The asbestos industry and its key supporters, such as Premier Charest and Prime Minister Harper, would likely have never changed. This is the same in most struggles for social justice. The key wrongdoers rarely abandon their vested interests. However, unless it is a dictatorship, the wrongdoers depend on social complicity. Enormous wrongdoing succeeds only when societies permit it by their silence.

Wrongdoing is enabled, in particular, by those who have a responsibility to defend the public good but fail to do so. By turning a blind eye, they hide the wrongdoing. The campaign did not allow leading figures to hide. It put the spotlight on political leaders, union leaders, prestigious health organizations, top public servants responsible for health, Quebec’s top two English-speaking universities, journalists, academics, and students. It challenged them to stop being complicit with wrongdoing and to speak out publicly to condemn the scientific deception and human harm caused by the asbestos industry.

Many showed courage and integrity and stopped being complicit. Starting with just a small number—a tiny handful of scientists, just one or two academics, just one political leader, just one union leader, just one journalist—they started a movement that became unstoppable.

Again and again, we see how vested interests—whether asbestos, tobacco, the fossil fuel industry, hazardous chemicals, the sugar industry—distort scientific evidence and corrupt public policy so that it serves industry profits instead of the public good. Industry lobby groups aggressively attempt to intimidate and silence independent scientists whose work is inconvenient for industry profits.

It is essential that scientists not be silenced and fulfill their responsibility to defend scientific integrity and evidence-based public policy.

## 4. Conclusions

The asbestos battle in Quebec demonstrates how a determined advocacy campaign involving international solidarity, the scientific community, activists, and asbestos victims succeeded, against all odds, in defeating the asbestos industry and changing public health policy in Quebec and Canada. As a result, instead of the planned expansion of asbestos mining and export, Canada is now in the process of legislating a ban on the mining, use, and export of asbestos. Instead of being a propagandist for the asbestos industry, Canada is now an ally in efforts to achieve a global asbestos ban.
